# Comparison of SD Bioline Malaria Ag Pf/Pan and Acro Malaria P.f./P.v./Pan with Microscopy and Real Time PCR for the Diagnosis of Human *Plasmodium* Species

**DOI:** 10.3390/diagnostics14070721

**Published:** 2024-03-29

**Authors:** Marylin Madamet, Isabelle Fonta, Joel Mosnier, Nicolas Benoit, Rémy Amalvict, Sébastien Briolant, French National Reference Centre for Imported Malaria Study Group, Bruno Pradines

**Affiliations:** 1 Unité Parasitologie et Entomologie, Département de Microbiologie et Maladies Infectieuses, Institut de Recherche Biomédicale des Armées, 13005 Marseille, France; mmadamet@gmail.com (M.M.); isabelle.fonta.09@gmail.com (I.F.); joelmosnier@orange.fr (J.M.); nicobenoit73@hotmail.com (N.B.); remy_alt@yahoo.fr (R.A.); sbriolant@wanadoo.fr (S.B.); 2 Aix Marseille Univ, SSA, AP-HM, RITMES, 13005 Marseille, France; 3 IHU Méditerranée Infection, 13005 Marseille, France; 4 Centre National de Référence du Paludisme, 13005 Marseille, France

**Keywords:** malaria, *Plasmodium*, diagnosis, rapid diagnostic test, microscopy, real-time PCR

## Abstract

The early diagnosis of malaria is crucial to controlling morbidity and mortality. The World Health Organization (WHO) recommends diagnosing malaria either using light microscopy or a malaria rapid diagnostic test (RDT). Most RDTs use antibodies to detect two *P. falciparum* histidine-rich proteins named PfHRP2 and PfHRP3. However, false-negative results are known to occur due to the poor performance of RDTs depending on the species and the deletion of the *Pfhrp2* and *Pfhrp3* genes. This study evaluated new malaria RDTs for the detection of the human *Plasmodium* species. The Acro Malaria P.f./P.v./Pan Rapid Test Cassette allows the qualitative detection of parasite antigens, such as PfHRP2 specific to *Plasmodium falciparum*, PvLDH specific to *Plasmodium vivax*, and/or panLDH *Plasmodium* genus lactate dehydrogenase, in the blood of infected individuals. This RDT was assessed against 229 samples collected from imported malaria cases, mainly from Africa. The samples were previously diagnosed using light microscopy and RDT (SD Malaria Ag P.f./Pan, SD Bioline Alere Abbott), then confirmed using real time PCR. The two RDTs were evaluated using a comparison with real time PCR as the reference method, and their performances were compared with each other. Compared to SD RDT, the Acro RDT showed a better sensitivity to *P. falciparum* (96.8% vs. 89.8%), *P. vivax* (78.6% vs. 64.3%), *P. ovale* (73.7% vs. 5.3%), and *P. malariae* (20.0% vs. 0%). The respective specificities of the Acro RDT and SD RDT are 90.7% vs. 95.3% to *P. falciparum*, 100% to *P. vivax*, and 100% vs. 100% to *Plasmodium* genus. Therefore, Acro RDT showed better performance in the identification of *P. ovale* and low parasitaemia of *P. falciparum*. In addition, Acro RDT has the advantage of detecting PvLDH-specific antigens. The Acro Malaria RDT presents the benefits of detecting a *P. falciparum* antigen (PfHRP2) and a *P. vivax* antigen (PvLDH) with high sensitivity (96.8% and 73.7%, respectively) and specificity (90.7% and 100%, respectively). Acro Malaria P.f./P.v./Pan rapid diagnostic tests could be effectively used in endemic areas, especially when microscopic examination cannot be performed.

## 1. Introduction

Malaria remains a significant cause of morbidity and mortality in developing countries. In 2022, the number of malaria deaths was estimated at 608,000 and the number of clinical cases at 249 million [1].

Human malaria is caused by five species of the *Plasmodium* genus: *P. falciparum*, *P. vivax*, *P. ovale*, *P. malariae*, and *P. knowlesi*. *P. falciparum* and *P. vivax* are both species that can cause severe complications. *P. falciparum* is lethal, and is responsible for severe disease pathology and the majority of deaths due to malaria, especially in sub-Saharan Africa. *P. vivax* can cause severe, even fatal infections, and is highly prevalent across South America [2]. *P. ovale* and *P. malariae* remain tropical diseases of lower severity compared to falciparum malaria, and their prevalence has increased particularly in endemic areas where *P. falciparum* is in decline [3,4]. Most *P. knowlesi* infections appear to be benign with low parasitaemia, and have mainly been detected in South East Asia [5]. But deaths due to *P. knowlesi* infection were observed.

Africa is the continent which is most affected by malaria, and 80% of all malaria-related deaths are among children under the age of five. Since 2015, the WHO European Region has been free of malaria. The French National Reference Centre for Malaria (Malaria CNR) is responsible for epidemiological surveillance of imported malaria cases in France (2322 cases in 2021) and among French military personnel [6]. French military personnel are exposed to *P. vivax* in French Guiana where *P. vivax* is predominant and co-exists with *P. falciparum* [7].

To avoid the morbidity and mortality associated with malaria, effective diagnostic techniques and treatment are essential. Diagnosis using microscopy (thin blood films and thick blood smears) is recommended as the gold standard by the World Health Organization (WHO) [1]. Malaria rapid diagnostic tests (RDTs) are an alternative to microscopic diagnosis, and remain dominant on the market, with variations in targets and formats. An RDT is a way of easily and rapidly diagnosing malaria, especially when microscopic examination cannot be performed. Malaria RDTs detect malaria antigens in the blood of infected individuals, such as the HRP2 antigen expressed by *P. falciparum*, and LDH test lines to detect multiple other species [8].

Nevertheless, RDTs fall short when it comes to detecting low parasitaemia, mixed infections, species other than *P. falciparum*, and *Pfhrp2*-deleted *P. falciparum*. It is thus essential to select the most effective kit depending on test performance (sensitivity and specificity) [9].

The aim of this study was to evaluate the sensitivity and specificity of new malaria RDTs marketed to detect the human *Plasmodium* species, namely the Acro Malaria P.f./P.v./Pan Rapid Test Cassette.

## 2. Materials and Methods

### 2.1. Sample Collection

The samples were collected from patients hospitalised in French hospitals between July 2021 and January 2023. The patients presented with imported malaria cases from endemic countries and their samples were sent to the French National Reference Centre for Malaria (CNR) (Institut de Recherche Biomédical des Armées, IHU Méditerranée Infection, Marseille). In this study, 97% of isolates were imported from African countries (Angola *n* = 1, Benin *n* = 4, Burkina Faso *n* = 10, Cameroon *n* = 27, Central African Republic *n* = 12, Chad *n* = 18, Comoros *n* = 20, Congo *n* = 10, Djibouti *n* = 3, Ethiopia *n* = 1, Ivory Coast *n* = 46, Gabon *n* = 9, Ghana *n* = 1, Guinea *n* = 22, Guinea Conakry *n* = 1, Madagascar *n* = 2, Mali *n* = 8, Mauritania *n* = 1, Niger *n* = 4, Nigeria *n* = 2, Senegal *n* = 10, Somalia *n* = 1, South Africa *n* = 1, Togo *n* = 8). Other isolates are imported from South America (Guyana *n* = 4, Mexico *n* = 1) and Oceania (Papua New Guinea *n* = 2). A total of 229 isolates were evaluated using PCR diagnosis, including 174 *P. falciparum*, 11 *P. vivax*, 14 *P. ovale*, nine *P. malariae*, 13 mixed infections (3 *P. falciparum*/*P. vivax*, 4 *P. falciparum*/*P. ovale*, 5 *P. falciparum*/*P. malaria*, and 2 *P. malaria/P. ovale*) and 8 negative samples.

### 2.2. Malaria Diagnosis Using Microscopy

The samples were previously diagnosed using microscopy as soon as they were received at the French Malaria CNR laboratory.

Thin blood smear was prepared from peripheral venous blood collected in Vacutainer^®^ ACD tubes (Becton Dickinson, Rutherford, NJ, USA) prior to patient treatment. The slides were stained by eosin and methylene blue using a RAL^®^ kit (Réactifs RAL, Paris, France). Stained blood films were examined using certified operators to determine parasite density and to confirm species-specific mono-infection or mixed infections. The parasitaemia percentage was estimated by counting the number of infected cells as a percentage of red blood cells. Samples were considered negative if no parasite was found after examination of 100 fields (>20 min of examination and around 100,000 erythrocytes observed).

### 2.3. Malaria Diagnosis by RDTs

Samples were diagnosed using RTDs available on the market, including the SD BIOLINE Malaria Ag P.f/Pan (Cat. No. 05FK63, SD Bioline Alere Abbott, Standard Diagnostics, Seoul, Republic of Korea) and the Acro Malaria P.f./P.v./Pan Rapid Test Cassette (Cat. No. IMPVF-402, Acro Biotech, Montclair, CA, USA).

The RDT kit used by the Malaria CNR at the time of the study was the SD BIOLINE Malaria Ag P.f/Pan. This test specifically targets the histidine-rich-protein 2 (PfHRP2) expressed by *P. falciparum* and common *Plasmodium* lactate dehydrogenase (panLDH) of *Plasmodium* species in human whole blood. The performance of the SD BIOLINE RDT stated by the manufacturer is a sensitivity of 99.7% and a specificity of 99.5% for *P. falciparum* (PfHRP-2) and a sensitivity of 95.5% and a specificity of 99.5% for pan (panLDH).

To determine the best performance between the two RDTs, the Acro Malaria RDT marketed in France is used. This test qualitatively detects four *Plasmodium* human species in whole blood with specific antigens for *P. falciparum* (PfHRP2), *P. vivax* lactate dehydrogenase (PvLDH), and *Plasmodium* lactate dehydrogenase (panLDH), expressed by all human malaria species. The performance of this RDT stated by the manufacturer is 98.7% in relative sensitivity and 99.3% in relative specificity.

RDTs were performed according to each manufacturer’s instructions.

### 2.4. Malaria Diagnosis by Real Time PCR

The species diagnostics were validated using real-time PCR on a Light Cycler 2.0 (Roche Group, Basel, Switzerland) for the identification of four human *Plasmodium* species, as previously described [10]. Briefly, DNA was isolated from 200 µL of whole blood using the QIAamp^®^ DNA Blood Mini kit (Qiagen, Hilden, Germany), as recommended by the manufacturer, which was followed by individual real time PCR. Each isolate was detected by targeting a specific gene for each of four human Plasmodium species using the Light Cycler^®^ TaqMan^®^ Master Mix (Roche Group, Switzerland). For each PCR run, two negative controls (water and human DNA) and a positive control (DNA from each species) were used.

### 2.5. Statistical Analysis

With real-time PCR as the reference method, the statistical analysis compared the performance results of the Acro Malaria P.f./P.v./Pan Rapid Test against the SD Malaria Ag Pf/Pan test.

To evaluate test performances, the sensitivity and specificity were calculated using the following formula (TP = true positives, FN = false-negative, FP = false-positive, and TN = true negative):

Sensitivity = TP/(TP + FN), proportion of samples with the malaria species correctly identified.
Specificity = TN/(FP + TN), proportion of samples without the malaria species correctly identified.

Confidence intervals (95% CI) for sensitivity and specificity are evaluated using the Wilson score method [11].

RDT performance was thus calculated compared with real time PCR results with 95% CI for the following values: sensitivity, specificity, positive predictive value (PPV), and negative predictive value (NPV).

To compare the two RDTS, the Kappa values were calculated with a 95% confidence interval (CI). The Kappa results were interpreted as follows: values ≤ 0 as indicating no agreement, 0.01–0.20 as none to slight, 0.21–0.40 as fair, 0.41–0.60 as moderate, 0.61–0.80 as substantial, and 0.81–1.00 as almost perfect agreement [12].

## 3. Results

All the samples were successively diagnosed using light microscopy and RDTs (SD Malaria Ag Pf/Pan and SD Bioline Alere Abbott), then confirmed using real-time PCR.

Diagnosis using real-time PCR served as the reference method, as this molecular diagnostic technique has higher sensitivity and specificity and makes it possible to detect low parasitaemia.

**Figure 1:**
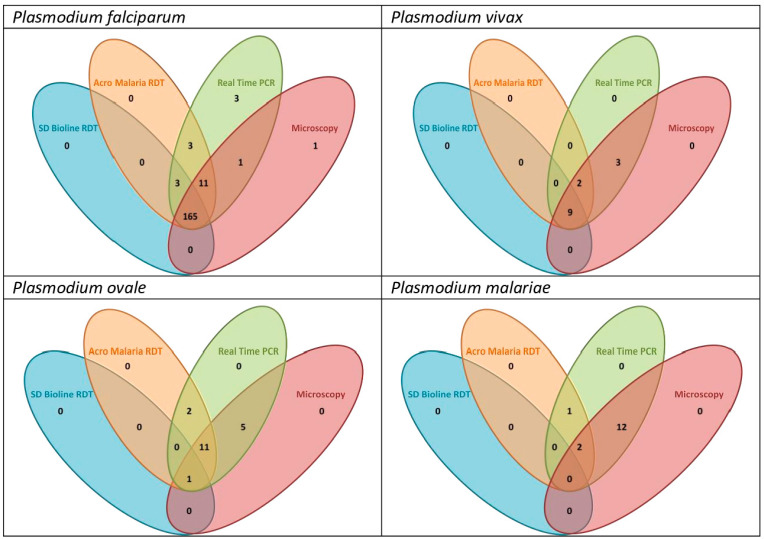
Venn diagrams showing the similarities and discrepancies between different malaria diagnostic methods with real-time PCR as the reference method.

Venn diagrams show a summary of the relationships between the malaria diagnostic methods among the four malaria species (Figure 1).

### 3.1. Microscopy

All samples were detected using thin blood smears and confirmed using real-time PCR (Table 1).

Using PCR as the reference, the majority (89.5%) of the isolates were microscopically identified for malaria. Mixed infections were difficult to identify using microscopy. Of the eighteen isolates microscopically tested as negative, eight were confirmed to be positive using real-time PCR as *P. falciparum*, one as *P. malariae*, and one as *P. vivax*.

The level of parasitaemia ranged from 0.001% to 35%. A total of 229 isolates were evaluated, including 174 *P. falciparum* (parasitaemia from 0.001% to 35%), 11 *P. vivax* (parasitaemia from 0.01% to 0.3%), 14 *P. ovale* (parasitaemia from 0.001% to 0.3%), 9 *P. malariae* (parasitaemia from 0.001% to 0.25%), and 8 negative samples.

### 3.2. Comparison of Malaria RDTs

A total of 229 PCR-tested samples were evaluated with Acro Malaria and SD BIOLINE RDTs. Respectively, the number of *P. falciparum*-positive tests was 180/186 (96.8%) and 167/186 (89.8%), 11/14 (78.6%) and 9/14 (64.3%) for *P. vivax*-positive, 14/19 (73.7%) and 1/19 (5.2%) for *P. ovale*-positive, and 3/15 (20%) and 0/15 (0%) for *P. malariae*-positive (Table 2).

Compared to Acro Malaria, the SD BIOLINE RDT did not perform well, with a “fair” agreement, Kappa = 0.21 (95% CI 15.9–26.3%) for PfHRP2 specific of *P. falciparum* and a “substantial” agreement, Kappa = 0.62 (95% CI 53.1–70.9%), for panLDH specific to the *Plasmodium* genus.

Among the negative-RDT samples, 18 Acro Malaria RDT (7.9%) and 44 SD BIOLINE RDT (19.2%) were false-negatives based on PCR (Table 3). However, six negative-Pf/Pan RDT isolates were found to be Pf-positive using PCR. These isolates come from African countries (Central African Republic, Ivory Coast, Comoros, Cameroon, and Mali) and the last from Afghanistan. This study also highlighted that RDT-positive samples (three Acro Malaria RDT and tqo SD BIOLINE RDT) were found to be negative-PCR but corresponded to the detection of residual HRP2 (Table 3). In addition, 13 isolates were mixed infections on PCR; all 13 isolates were detected using Acro Malaria RDT, while only 6 samples were detected using SD BIOLINE RDT.

Based on the parasitemia level, the samples were classified into four categories: 0.001 ≤ *p* < 0.01, 0.01 ≤ *p* < 0.1, 0.1 ≤ *p* < 1, and *p* ≥ 1 (Table 3). The number of positive-RTD samples was calculated based on detecting PfHRP2-, PvLDH-, or panLDH-specific antigens and parasitemia-level categories. The performance of RTD does not appear to be varied according to parasitemia level, except for samples of low parasitaemia, which are less well-detected as predictable.

The Acro Malaria RDT showed high-quality performance, identifying the four human *Plasmodium* species (*P. falciparum*, *P. vivax*, *P. ovale*, and *P. malariae*) and, more particularly, *P. falciparum* with low parasitaemia and *P. ovale*. Moreover, this RDT also specifically differentiates between *P. vivax* infections with good performance, due to the presence of the PvLDH specific antigen. However, no difference in sensitivity to *P. vivax* was found between the panLDH or PvLDH bands.

Both RDTs performed poorly in detecting *P. malariae*, resulting in poor diagnosis of imported *P. malariae* cases.

### 3.3. Performance of Malaria RDTs

With respect to performances, the results of this study showed best sensitivity and best specificity of the Acro Malaria RDT compared to the SD BIOLINE RDT used by the Malaria CNR. The sensitivities of the Acro Malaria RDT (vs. SD BIOLINE RDT) for each respective malaria species were 96.8% (95% CI 93.1–98.5%) to *P. falciparum* (vs. 89.8–95% CI 84.6–98.7%), 78.6% (95% CI 52.4–92.4%) to *P. vivax* (vs. 64.3–95% CI 38.8–83.6%), 73.7% (95% CI 51.2–88.2%) to *P. ovale* (vs. 5.3–95% CI 0.9–24.6%), and 20% (95% CI 7.1–45.2%) to *P. malariae* (vs. 0–95% CI 0–0.2%) and the respective specificities 90.7% (95% CI 78.4–96.3%), PPV 97.8% and NPV 86.7% to *P. falciparum* (vs. 95.3–95% CI 84.5–98.7%–PPV 98.8% and NPV 68.3%), 100% (95% CI 98.2–100%), PPV 100% and NPV 98.6% to *P. vivax* and 100% (95% CI 67.6–100%), and PPV 100% and NPV 10.5% to *Plasmodium* genus (vs. 100–95% CI 67.6–100%–PPV 100% and NPV 6.5%) (Table 4).

This study made it possible to observe a variation of sensitivity between malaria RDTs on the market based on Ct cut-off values. On the basis of Ct values, real time PCR positive samples were divided into three categories: low Ct < 25, medium Ct 25–30 and high Ct 30–40. The sensitivity of RDTs was calculated according to malaria species and Ct value categories. There was indeed a relationship between sensitivity and Ct values: the sensitivity of the ACRO Malaria RDT can reach 100% for *P. falciparum*, *P. vivax*, and *P. ovale* for Ct values less than 25, and negative sensitivities can be obtained at Ct values greater than 30. Ct values < 25 are likely to be associated with high parasitaemia, and therefore RDTs perform better at these Ct values. However, RDTs perform well with medium Ct values (Table 5).

**Figure 2:**
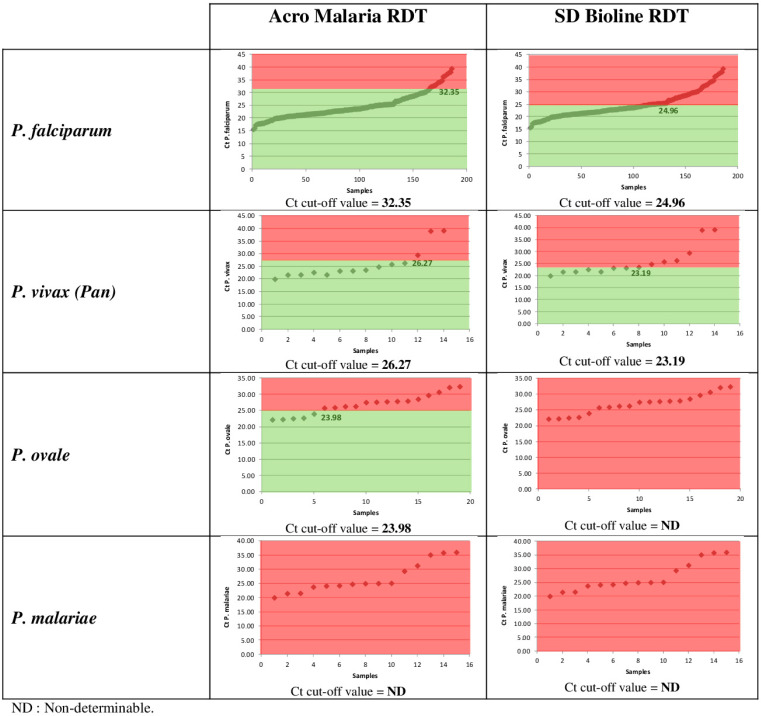
Sensitivity of RDTs according to the optimal Ct cut-off value.

Considering the Ct value intervals, sensitivities are better for the ACRO Malaria RDT than the SD BIOLINE RDT. The optimal Ct cut-off values that maximised sensitivity were 32.35 for *P. falciparum*, 26.27 for *P. vivax*, and 23.98 for *P. ovale*, using ACRO Malaria RDT (Figure 2).

## 4. Discussion

The severity of malaria requires early management and therapeutic treatment involving rapid and accurate diagnosis. Although microscopy diagnosis remains the gold standard for the investigation of malaria, the WHO recommends that all suspected malaria cases are diagnosed using RDT.

Currently, more than 200 different malaria RDTs are commercially available, based on the detection of parasite proteins using immunochromatography [13]. A complete RDT can be performed in less than 15 min. The test is easy to use, cost effective, and can be used in the field for malaria diagnosis.

New malaria RDTs were evaluated for the detection of the human *Plasmodium* species, namely the Acro Malaria P.f./P.v./Pan Rapid Test Cassette. The Acro Malaria RDT makes it possible to specifically detect PfHRP2 *P. falciparum*, of PvLDH *P. vivax*, and panLDH *Plasmodium* genus lactate dehydrogenase for all species. PfHRP2- and PvLDH-based RDTs are more sensitive for the detection of *P. falciparum* and *P. vivax*, respectively, than PanLDH-based RDTs.

According to the manufacturer, the performance of this RDT is 98.7% in relative sensitivity and 99.3% in relative specificity. To confirm the appropriate performance of the RDTs, 229 samples collected from imported malaria were evaluated using Acro Malaria RDT. Compared to SD BIOLINE RDT, the Acro RDT showed a better sensitivity to *P. falciparum* (96.8% versus 89.8%), *P. vivax* (78.6% versus 64.3%), *P. ovale* (73.7% versus 5.3%), and *P. malariae* (20.0% versus 0%). This RDT showed specificities of 90.7% for *P. falciparum*, 100% for *P. vivax*, and 100% for *Plasmodium* genus.

RDTs have higher sensitivity at Ct values < 25, probably associated with elevated parasitaemia. The optimal Ct cut-off values for maximum sensitivity are better with the Acro Malaria RDT.

In the malaria sample with very low parasitaemia (<0.001%) and mixed malaria infection, the RDT is less reliable [14]. The Acro Malaria RDT showed better performance at identifying low parasitaemia of *P. falciparum* and mixed malaria infections, compared to the SD BIOLINE RDT. Even if the sensitivity of RTDs is affected by low parasitic densities (*p* < 0.001%), the performance of evaluated RTDs remains correct according to parasitemia levels.

In cases of *P. malariae* malaria, misdiagnosis is linked to the poor performance of RDTs, underlined in the majority of manufactured RDT [15].

Every year, the French Malaria CNR diagnoses imported malaria cases in France and cases observed in the French armed forces. Members of the French military are deployed in parts of Africa where *P. falciparum* is predominant, but also in parts of the continent where the transmission of *P. ovale* has been reported, such as the Ivory Coast, Gabon, and Senegal [16,17,18]. Between 2000 and 2015, 465 cases of *P. ovale* (7.2% of all malaria cases) were reported in French soldiers [19,20]. Moreover, many military operations are performed every year in French Guiana, where *P. vivax* is endemic and predominant [21]. Between 2000 and 2015, 1877 cases of *P. vivax* (29% of all malaria cases) were reported in French soldiers [19,20]. Consequently, it is important to identify RDTs which are effective at detecting all species. The low performance of the diagnosis of *P. vivax* using commercialised RDTs is due to a lower parasite density in *P. vivax* infections and lower expression of the specific antigen. By selecting the RTD on the market with the best performance, it is possible to reduce the number of undiagnosed *P. vivax* clinical cases [22]. Consequently, the use of Acro Malaria RDT to specifically detect PvLDH is fundamental. Furthermore, this test showed the best performance at identifying *P. ovale*.

However, RDT performances are influenced by numerous factors, leading to false results.

False-positive results could be explained by the persistence of parasite PfHRP2 in the circulation after parasite clearance. In this study, three RDT-positive samples were negative with qPCR. These samples are from patients who remain RDT-positive for several weeks after parasite treatment, due to the detection of residual HRP2 [23].

False-negative results are reported with PfHRP2-based RDTs due to (i) low-parasite density infections, (ii) a prozone-like effect in high parasite densities, and (iii) modified epitope PfHRP2 by genetic polymorphism of the *Pfhrp*2/3 gene or by *Pfhrp2* gene deletion. In the sub-Saharan African countries, *pfhrp2* deletions have recently been reported through the surveillance WHO program. The false-negative-Pf/Pan RDT isolates of this study were identified in the African countries (Central African Republic, Ivory Coast, Comoros, Cameroon, and Mali) where the presence of parasites with *pfhrp2* deletions has not been evaluated or reported with a low prevalence [24,25]. All these samples were *pfhrp2*-deleted *P. falciparum* parasites (unpublished data) as a negative result for *pfhrp2* PCR.

The prevalence of *Pfhrp2* gene deletion has been well documented in *P. falciparum* isolates [23,24,25,26,27,28,29,30]. The *Pfhrp2*-deleted parasites are capable of inducing malaria, and may significantly reduce the effectiveness of RDTs. Consequently, many *P. falciparum* infections might remain undiagnosed and untreated, causing a circulation of *Pfhrp2*-deleted strains and causing the disease to persist in the population. The number of negative-RDT samples is higher with SD BIOLINE RDT than the Acro Malaria RDT [9].

## 5. Conclusions

Based on all these arguments, Acro Malaria P.f./P.v./Pan Test Cassette has proven to be effective in replacing the SD BIOLINE Malaria Ag P.f/Pan for the diagnosis of human *Plasmodium* species.

## Figures and Tables

**Table 1:** Thin blood smears compared with real-time PCR results.

Malaria Diagnosis	*P. falciparum*	*P. vivax*	*P. ovale*	*P. malariae*	Non-Identified Infections ^a^	Negative
Microscopy	166	11	12	8	14	18
Real time PCR	174	11	14	9	13	8

^a^ The non-identified infections included 13 mixed infections and 1 sample with significant haemolysis.

**Table 2:** Comparison of the ACRO MALARIA and SD BIOLINE RDT with real-time PCR.

Malaria Diagnosis	*P. falciparum* ^a^	*P. vivax* ^a^	*P. ovale* ^a^	*P. malariae* ^a^	Negative
Real-time PCR	186	14	19	15	8
ACRO MALARIA RDT	180	11	14	3	34
SD BIOLINE RDT	167	9	1	0	65

^a^ Including the 13 mixed infections.

**Table 3:** RDT results by parasitemia category.

RDTs	Specific Antigen	Parasitemia Category (%)				Negative-RDT	False-Negative	False-Positive
		0.001 ≤ *p* < 0.01	0.01 ≤ *p* < 0.1	0.1 ≤ *p* < 1	*p* ≥ 1			
ACRO RDT	PfHRP2 (*n* = 186)	20	42	70	48	6	4	3
	PvLDH (*n* = 14)	0	5	6	0	3	1	0
	panLDH (*n* = 229)	4	28	71	47	79	13	0
SD RDT	PfHRP2 (*n* = 186)	12	38	69	48	19	18	2
	panLDH (*n* = 229)	2	12	45	46	124	26	0

**Table 4:** Sensitivity and specificity of ACRO MALARIA and SD BIOLINE RDT.

*Plasmodium* Species	Sensitivity ^a^ Acro Malaria RDT	Specificity ^b^ Acro Malaria RDT	PPV ^c^ Acro Malaria RDT	NPV ^d^ Acro Malaria RDT	Sensitivity ^a^ SD Bioline RDT	Specificity ^b^ SD Bioline RDT	PPV ^c^ SD Bioline RDT	NPV ^d^ SD Bioline RDT
*P. falciparum* (*n* = 186)	96.8%	90.7%	97.3%	86.7%	89.8%	95.3%	98.8%	68.3%
*P. vivax* (*n* = 14)	78.6%	100.0%	100%	98.6%	64.3%	ND	ND	ND
*P. ovale* (*n* = 19)	73.7%	ND	ND	ND	5.3%	ND	ND	ND
*P. malariae* (*n* = 15)	20.0%	ND	ND	ND	0%	ND	ND	ND
*Plasmodium*	69.2%	100.0%	100%	10.5%	47.5%	100.0%	100%	6.5%

^a^ Sensitivity: TP/(TP + FN), ^b^ Specificity: TN/(FP + TN), ^c^ positive predictive value: PPV, ^d^ negative predictive value: NPV, ND: non-determinable.

**Table 5:** Sensitivity of RDTs by Ct value intervals.

RDTs	Species	Cycle Threshold Category		
		Sensitivity at Ct < 25	Sensitivity at Ct 25–30	Sensitivity at Ct > 30
ACRO RDT	*P. falciparum* (*n* = 186)	100	100	73.9
	*P. vivax* (*Pv*) (*n* = 14)	100	66.7	0
	*P. vivax* (*Pan*) (*n* = 14)	88.9	66.7	50
	*P. ovale* (*n* = 19)	100	72.7	33.3
	*P. malariae* (*n* = 15)	12.5	0	50
SD RDT	*P. falciparum* (*n* = 186)	99.1	85.1	52.2
	*P. vivax* (*Pan*) (*n* = 14)	88.9	33.3	0
	*P. ovale* (*n* = 19)	20	0	0
	*P. malariae* (*n* = 15)	0	0	0

Sensitivity: TP/(TP + FN).

## Data Availability

The datasets analysed in this study are available from the corresponding author on reasonable request.
